# Non-invasive assessment of oxygenation status using the oxygen reserve index in dogs

**DOI:** 10.1186/s12917-023-03804-z

**Published:** 2023-11-18

**Authors:** Francesca Zanusso, Giulia Maria De Benedictis, Polina Zemko, Luca Bellini

**Affiliations:** Department of Animal Medicine, Productions and Health, University of Padova, Legnaro, Padova, 35020 Italy

**Keywords:** Oxygen reserve index, Dog, Arterial partial pressure of oxygen, Pulse oximetry, Hyperoxaemia

## Abstract

**Background:**

The oxygen reserve index (ORi) is a real-time, continuous index measured with multi-wavelength pulse CO-oximetry technology. It estimates mild hyperoxemia in humans, which is defined as a partial pressure of oxygen (PaO_2_) level between 100 and 200 mmHg. The objectives of this study were to assess the correlation between ORi and PaO_2_, as well as to determine its ability in detecting mild hyperoxemia in dogs.

**Methods:**

This prospective observational study enrolled 37 anaesthetised and mechanically ventilated dogs undergoing elective procedures. Simultaneous measurements of ORi and PaO_2_ were collected, using a multi-wavelength pulse CO-oximeter with a probe placed on the dog’s tongue, and a blood gas analyser, respectively. A mixed-effects model was used to calculate the correlation (r^2^) between simultaneous measurements of ORi and PaO_2_. The trending ability of ORi to identify dependable and proportional changes of PaO_2_ was determined. The diagnostic performances of ORi to detect PaO_2_ ≥ 150 mmHg and ≥ 190 mmHg were estimated using the area under the receiver operating characteristic curve (AUROC). The effects of perfusion index (PI), haemoglobin (Hb), arterial blood pH and partial pressure of carbon dioxide (PaCO_2_) on AUROC for PaO_2_ ≥ 150 mmHg were evaluated.

**Results:**

A total of 101 paired measurements of ORi and PaO_2_ were collected. PaO_2_ values ranged from 74 to 258 mmHg. A strong positive correlation (r^2^ = 0.52, *p* < 0.001) was found between ORi and PaO_2_. The trending ability ORi was 90.7%, with 92% sensitivity and 89% specificity in detecting decreasing PaO_2_. An ORi value ≥ 0.53 and ≥ 0.76 indicated a PaO_2_ ≥ 150 and ≥ 190 mmHg, respectively, with ≥ 82% sensitivity, ≥ 77% specificity and AUROC ≥ 0.75. The AUROC of ORi was not affected by PI, Hb, pH and PaCO_2_.

**Conclusions:**

In anaesthetised dogs, ORi may detect mild hyperoxaemia, although it does not replace blood gas analysis for measuring the arterial partial pressure of oxygen. ORi monitoring could be used to non-invasively assess oxygenation in dogs receiving supplemental oxygen, limiting excessive hyperoxia.

## Background

In anaesthetised veterinary patients, assessment of oxygenation is recommended from induction through the recovery period, and clinically relevant decreasing of arterial oxygen content can be monitored by pulse oximetry whenever possible [1]. The pulse oximeter helps detecting hypoxaemia, estimating the percent of arterial haemoglobin saturated with oxygen in the periphery (SpO_2_). However, SpO_2_ provides limited information in hyperoxaemic patients, remaining > 98% for arterial partial oxygen pressure (PaO_2_) > 100mmHg [2]. Measuring hyperoxaemia in anaesthetised veterinary patients is crucial to titrate oxygen administration and prevent the adverse effect of elevated inspired fraction of oxygen (FiO_2_), such as absorption atelectasis [3, 4]. To date, hyperoxaemia in dogs can be detected using arterial blood gas analysis, that measures PaO_2_, but the technique is invasive and provides intermittent and delayed information about oxygenation status. Arterial blood sampling can be challenging particularly in small dogs, and could lead to vascular infection [5].

The oxygen reserve index (ORi) is a new real-time continuous index, that estimates in humans the oxygen reserve, defined as PaO_2_ between 100 and 200 mmHg. This index ranges from 0 to 1, and is measured non-invasively with a multi-wave pulse CO-oximeter that detects haemoglobin absorption in both arterial and venous blood. At PaO_2_ above 100 mmHg, arterial oxygen saturation (SaO_2_) plateaus at 100%, while venous oxygen saturation (SvO_2_) continues to increase until PaO_2_ is 200 mmHg [6]. Thus, ORi can identify increases in SvO_2_ when SaO_2_ has already reached its plateau.

This multi-wave CO-oximeter offers the benefits of ease of use and continuous or long-term non-invasive monitoring. However, as with the traditional pulse oximeter, its accuracy is theoretically affected by patient-related factors such as skin pigmentation, severe anaemia, hypotension, hypothermia, low local perfusion, and ambient light, [7]. The peripheral perfusion on the measurement site is estimated by the perfusion index (PI), which is measured with ORi and SpO_2_ by the CO-oximeter. The value of PI recorded is based on the ratio of pulsatile to non-pulsatile blood flow on the site of the sensing probe application [8].

Although ORi cannot replace arterial blood gas analysis in humans, many studies reported a positive correlation with PaO_2_ [9–11]. However, ORi was poorly investigated in veterinary medicine. In a preliminary study in anaesthetised donkeys, ORi showed a mild correlation with PaO_2_ between 100 and 200 mmHg [12]. Additionally, the index was used to monitor the response to oxygen administration in sedated dogs, increasing as the fraction of inspired oxygen increases [13].

The aims of this study were to investigate the correlation between ORi and PaO_2_ in anaesthetised dogs, to study the trending ability of ORi in identifying dependent changes of PaO_2_, and to evaluate the diagnostic performance of ORi in detecting different thresholds of hyperoxaemia, defined as PaO_2_ ≥ 150 mmHg and PaO_2_ ≥ 190 mmHg. These values identify a safe range of PaO_2_ that avoids severe hyperoxaemia while preserving a safe level of arterial oxygen during anaesthesia [4, 10]. We hypothesized that ORi might detect hyperoxia also in anaesthetised dogs and that changes in ORi values over time positively correlate with consistent changes in PaO_2_.

## Results

### Animals

The study enrolled thirty-seven dogs between October 2022 and May 2023. In all dogs, an arterial catheter was inserted without complications. Dogs were of both sexes (20 males and 17 females), aged 98 (10–169) months old and weighing 29.0 (14.5–50.0) kg, with a median body condition score of 6 (3–9) out of 9 [14]. Twenty-four animals underwent surgical procedures, 13 dogs were anaesthetized for diagnostic procedures. No animal received inotropes or vasopressors during the anaesthesia, 4 dogs were treated with intravenous (IV) crystalloid bolus infused (5 mL/kg) over 10–15 min at least 30 min before arterial blood sampling. All animals completed the study without complications in the intra or postoperative period.

### ORi-PaO_2_ correlation

**Fig. 1:**
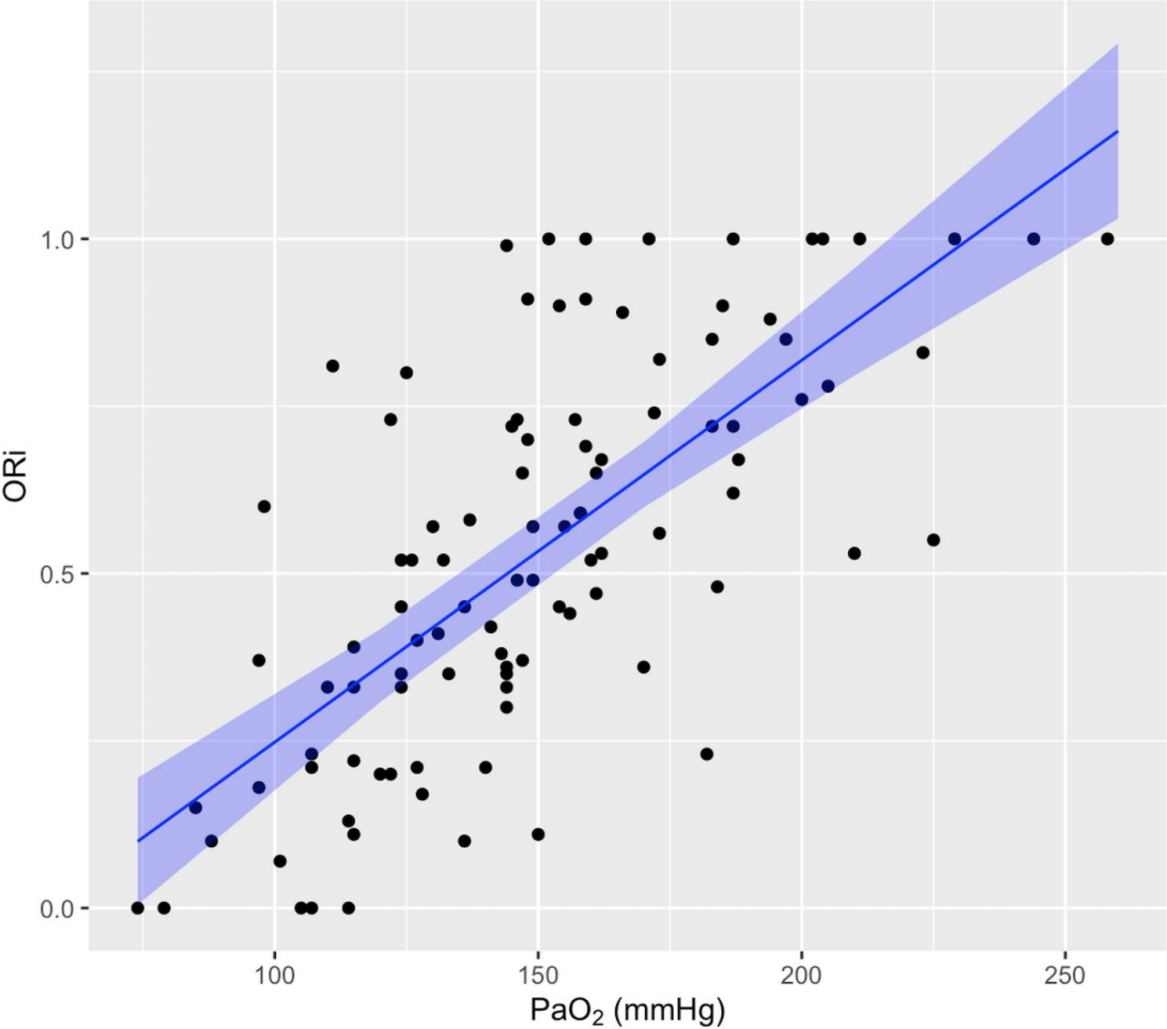
Mixed-effects model and correlation analysis between PaO_2_ and ORi, the blue area identify the 95% prediction intervals. Abbreviation: ORi Oxygen reserve index, PaO_2_ Arterial partial pressure of oxygen

A total of 101 matched ORi and PaO_2_ measurements were collected, with a median (range) of 3 (1–5) measurements from each dog. In 3 dogs, only one paired measurement was recorded. The parameters collected at each arterial sampling are shown in Table 1. A significant moderate linear positive correlation (r^2^ = 0.52–95% confidential interval 0.42–0.64, p < 0.001) between ORi and PaO_2_ values was found (Fig. 1; Table 2). In 3 of 5 data pairs, an ORi value of 0.0 matched a PaO_2_ > 100 mmHg. In 91 of 96 measurements of ORi > 0.0, SpO_2_ remained above 98%.

**Table 1:** The variables collected at time of the arterial blood sampling. Normally distributed variables were expressed as mean ± standard deviation, while others were expressed as median (minimum–maximum)

Variable (unit)	
ORi	0.52 (0–1)
SpO_2_ (%)	99 (93–100)
PR (bpm)	95 ± 29
PI	1.30 (0.23–3.40)
Temp (°C)	36.4 ± 1.2
pH	7.31 ± 0.09
PaO_2_ (mmHg)	150 ± 37
PaCO_2_ (mmHg)	44.9 (29.3–64.3)
Hct (%)	36 (15–49)
Hb (g/dl)	12.2 (5.0-16.7)
Lac (mmol/L)	1.41 (0.30–9.07)
HCO_3−_ (mmol/L)	22 ± 2.8

ORi Oxygen reserve index, SpO_2_ Oxygen saturation, PR Pulse rate, PI Perfusion index, Temp Temperature, PaO_2_ Arterial partial pressure of oxygen, PaCO_2_ Arterial partial pressure of carbon dioxide,  Hct Haematocrit, Lac Lactate, HCO_3_- Bicarbonate, Hb Haemoglobin

**Table 2:** Results of the mixed-effects model describing the effects of PaO_2_ and confounding factors on oxygen reserve index

	Estimate	Standard Error	t value	P Value
Intercept	-0.58299	0.19981	-2.918	0.005
PaO_2_	0.00574	0.00058	9.976	< 0.001
PI	0.02545	0.03382	0.753	0.455
PaCO_2_	0.00328	0.00312	1.052	0.296
Hct	0.00218	0.00315	0.692	0.493

PaO_2_ Arterial partial pressure of oxygen, PI Perfusion index, PaCO_2_ Arterial partial pressure of carbon dioxide, Hct Haematocrit

### Trending ability of ORi

**Fig. 2:**
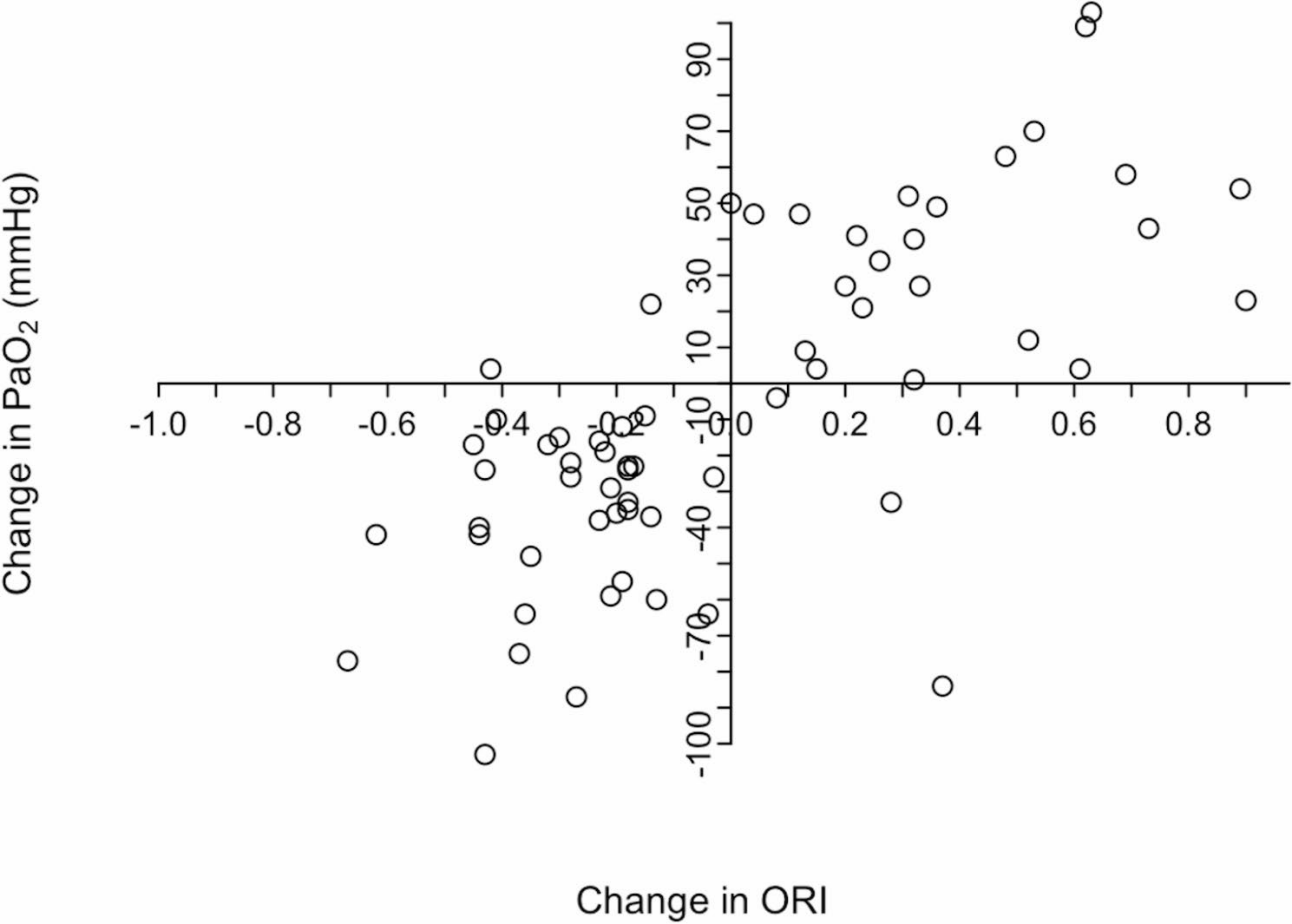
Four-quadrant plot used to analyse the trending ability of ORi in detecting dependent changes in PaO_2_. Abbreviation: ORi Oxygen reserve index, PaO_2_ Arterial partial pressure of oxygen

The concordance rate for the consecutive measurements of ORi and PaO_2_ (*n* = 64) was 90.7% (Fig. 2). ORi showed a sensitivity of 91.9% and a specificity of 88.9% in detecting decreasing PaO_2_.

### Diagnostic performance of ORi

**Fig. 3:**
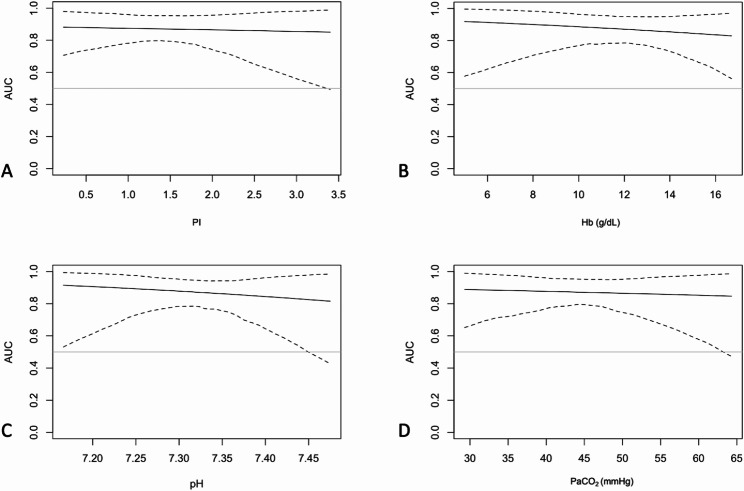
Effects of confounders on the diagnostic performance of oxygen reserve index. Variation of the area under the receiving operating characteristics curve (AUROC) for the ability of oxygen reserve index (ORi) to detect arterial partial pressure of oxygen (PaO_2_) ≥ 150 mmHg, over linear increases of perfusion index **(A)**, haemoglobin **(B)**, arterial blood pH **(C)**, arterial partial pressure of carbon dioxide **(D)**. The solid line represents the AUROC value based on the variable, and the dashed line represents the 95% CI. Abbreviation: PI Perfusion Index, Hb Haemoglobin (g/dl), PaCO_2_ Arterial partial pressure of carbon dioxide (mmHg)

The Youden index identified ORi cut-off values of 0.53 and 0.76, indicating PaO_2_ ≥ 150 mmHg and ≥ 190 mmHg, respectively, with AUROC (area under the ROC curve) ≥ 0.85 (Table 3). Diagnostic performance of ORi to detect PaO_2_ ≥ 150 mmHg did not appear to be affected by PI, Hb, pH and PaCO_2_ (Fig. 3). The graphs suggest that the AUROC remains relatively stable across varying variables. The 95% confidence interval reveals that, at the extremes, the limited number of observations amplifies the uncertainty in diagnostic performance.

**Table 3:** Diagnostic performance of oxygen reserve index (ORi) to detect arterial partial pressure of oxygen (PaO_2_) ≥ 150 mmHg and PaO_2_ ≥ 190 mmHg

Hyperoxaemia threshold	AUROC (CI 95%)	Youden Index	Best threshold	Sensitivity (CI 95%)	Specificity (CI 95%)	PPV (CI 95%)	NPV (CI 95%)
150 mmHg	0.85 (0.78–0.92)	0.59	0.53	0.82 (0.68–0.92)	0.77 (0.64–0.87)	0.74 (0.60–0.88)	0.84 (0.71–0.92)
190 mmHg	0.87 (0.76–0.97)	0.64	0.76	0.82 (0.48–0.98)	0.82 (0.72–0.89)	0.36 (0.24–0.84)	0.97 (0.88–0.99)

AUROC area under the receiver operating characteristic curve, PPV positive predictive value, NPV negative predictive value, CI confidential interval

## Discussion

This study investigated the correlation between ORi and PaO_2_ in dogs. The results suggest that ORi monitoring may effectively assess arterial oxygenation in anaesthetised and mechanically ventilated dogs inhaling a FiO_2_ above 0.21. In our study, ORi exhibited a moderate correlation with PaO_2_ values ranging between 74 and 258 mmHg. Similar findings have been reported in human patients, and various studies identified a linear correlation across a wide range of PaO_2_ values, differing from those presented by the manufacturer [9, 11, 15–17]. Furthermore, the ORi trending ability demonstrated a good concordance rate. The tested device exhibited acceptable sensitivity and specificity in detecting changes in ORi that corresponded consistently with alterations in PaO_2_. Consequently, in mechanically ventilated dogs, this novel index holds the potential to aid as an early warning indicator for impending deterioration in oxygenation status, before any significant changes in SpO_2_. Moreover, changes in ORi may be used to titrate oxygen administration to prevent severe hyperoxaemia.

As stated by the manufacturer in its current form, ORi is intended to serve as a “trend” variable rather than an absolute measurement of PaO_2_. In human medicine, the correlation between ORi and PaO_2_ within the ORi-sensitive range (i.e., PaO_2_ 100–200 mmHg) exhibited moderate to strong correlation, with r^2^ values from 0.53 to 0.82 [9, 11, 15–17]. In veterinary medicine, a preliminary study involving 28 anaesthetised donkeys indicated a mild correlation (Pearson correlation coefficient = 0.52, *p* < 0.001) in the same PaO_2_ range [12]. While the correlation between ORi and PaO_2_ tends to be stronger in dogs compared to donkeys, clinicians cannot depend solely on absolute ORi values for precise arterial oxygen measurements. In our study, ORi values of 0.0 and 1.0 did not consistently correspond to PaO_2_ levels ≤ 100 mm Hg and ≥ 200 mm Hg, respectively. Instead, we observed that relative changes in ORi within individual dogs yielded more clinically relevant information on oxygen status than the absolute ORi values. This observation is supported by the fact that when trending ability of ORi is evaluated, the concordance rate exceeding 90%. However, our findings do not provide enough evidence to suggest replacing the standard blood gas analysis method for measuring PaO_2_ with ORi. The limited reliability of ORi in estimating PaO_2_ is similar to that reported with standard pulse oximetry, which employs a similar technology based on light absorption. Comparable to ORi, SpO_2_ has also demonstrated an inability to effectively serve as a clinical surrogate for PaO_2_ in dogs [18].

The findings from our study have established ORi cut-off values that can predict minimal PaO_2_ levels. Values ≥ 0.53 and ≥ 0.76 reliably indicated PaO_2_ levels of ≥ 150 mmHg and ≥ 190 mmHg, respectively. In human patients, Applegate and colleagues (2016) noted that over 96% of ORi readings above 0.55 were associated with PaO_2_ values of 150 mmHg [9]. To prevent severe hyperoxia and minimize the risk of atelectasis in dogs during anaesthesia, it is advisable to maintain an FiO_2_ below 0.4, which typically results in a PaO_2_ of around 215 mmHg [4]. Maintaining ORi measurements within the range of 0.53 to 0.76 could help prevent excessive hyperoxaemia. The fraction of inspired oxygen might be adjusted to maintain ORi within this targeted range, thus mitigating adverse effects of intrapulmonary shunt. In human patients undergoing laparoscopic procedures, this index was successfully employed to limit intraoperative hyperoxaemia during gastrectomy [19].

Some patient-related factors, such as low local perfusion or severe anaemia, can impact the accuracy of pulse oximetry [7]. The perfusion index, which estimates local perfusion, did not demonstrate an effect on the ability of ORi in detecting PaO_2_ levels ≥ 150 mmHg. In our study, PI values showed relatively slight variation, ranging from 0.23 to 3.40. This observation may be attributed to the standardized positioning of the probe on the tongue, which could have contributed to the limited variability of PI among the readings. In a study involving 17 anaesthetised dogs with various pulse oximeter probe configurations, a wide spectrum of PI values was noted, spanning from 0.1 to 11.0, and a statistically significant difference in SpO_2_ was observed based on probe configuration [20].

Haemoglobin concentration did not influence the diagnostic performance of ORi in this study. In dogs, severe anaemia, indicated by a haematocrit lower than 10%, has been associated with reduced accuracy of SpO_2_ in estimating SaO_2_ determined by blood gas analysis [21]. However, none of the dogs included in this study were anaemic during the preoperative blood tests. The blood gas analyser consistently indicated a haematocrit level exceeding 15% for all patients, with the majority (75%) of measurements being above 31%.

Some dogs were hypothermic, which could have affected the measurements. However, the perfusion index in these animals remained within the range observed in anesthetized dogs with temperatures between 36 and 37 °C, indicating minimal impact on local perfusion [22]. Moreover, the effects of sedatives and anaesthetics on local perfusion were not taken into consideration in the current study. However, the relatively small range of PI, which did not affect the ability of ORi to detect PaO_2_ ≥ 150 mmHg, suggests that the variation in the anaesthetic protocol minimally impacted the signal acquisition.

Other factors that were considered as potential confounding variables, capable of affecting the diagnostic performance of ORi, included pH and PaCO_2_, with their potential to shift the haemoglobin dissociation curve, thus theoretically impairing the correlation between ORi and PaO_2_. However, these factors did not demonstrate any influence on ORi in our study. Factors influencing the accuracy of ORi measurements in humans have been poorly investigated. Nevertheless, a study involving critically ill patients confirmed that the diagnostic ability of ORi to detect PaO_2_ > 100 mmHg remained unaffected by variables such as PaCO_2_, haemoglobin levels, and PI [23]. In a few dogs, hypercapnia despite an end-tidal CO_2_ values below 45 mmHg was observed, which may indicate the development of intrapulmonary shunt [24]. Based on the results of the blood gas analysis, a single sigh recruitment manoeuvre was applied, and positive end-expiratory pressure of 4 cmH_2_O was applied.

Based on the blood gas results, lactatemia exceeded 3 mmol/L in four dogs, and in one animal, lactates exceeded 9 mmol/L. No hypotension or tachycardia was observed in these animals, and the capillary refill time was less than 2 s. The dogs received a crystalloid bolus as normal systemic haemodynamic might not match peripheral perfusion [25]. In these animals, the perfusion index remained between 1.6 and 2.4, indicating that at least in the tongue, perfusion was preserved.

Our study presents some limitations. Including the value of ORi obtained with PaO_2_ values outside the range suggested by the manufacturer may have influenced the correlation. Although most (83%) of PaO_2_ measurements (84/101) fell within the ORi-sensitive range, the overall PaO_2_ values extended from 74 to 258 mmHg. In human studies, the correlation between ORi and PaO_2_ remained statistically significant (*p* < 0.001) even for PaO_2_ values < 100 mmHg, but it appeared weak, with r^2^ ranging from 0.001 to 0.026 [9, 23]. Since the ORi-sensitive range has not been explored in dogs, we aimed to evaluate the relationship within PaO_2_ values from 100 to 200 mmHg. For this purpose, we maintained the FiO_2_ between 0.21 and 0.49. However, certain factors during anaesthesia, such as intrapulmonary shunt, might have decreased PaO_2_ values below the lower limit of PaO_2_ 100 mmHg. Regardless of this occurrence, we opted to include all paired measurements in the analysis, even those outside the sensing range, as such conditions could manifest in a clinical setting, and we would like to test the device in such a situation.

A further limitation is correlated to the canine haemoglobin, that may have interfered on the accuracy of our ORi measurements in this study. The readings of ORi values relies on a human-based algorithm, which could lead to inaccuracies when extrapolated to different species. Notably, haemoglobin in humans showed a dissociation curve distinct from that of haemoglobin in other species, including dogs [26]. In the context of veterinary medicine, a study involving 28 anesthetized donkeys revealed a low correlation between ORi and values of PaO_2_ ranging from 100 to 200 mmHg [12]. This observation could confirm the presence of species-specific disparities within the oxygen saturation haemoglobin curve.

Moreover, even though we aimed to standardize the positioning of the probe, an inadequate contact or alignment between the emitting and detecting part of the probe could not be completely ruled out. Utilizing a single size probe across dogs of varying weights could potentially lead to distinct ORi readings obtained from different areas of the tongue. Within canine subjects, evident fluctuations in SpO_2_ values have been observed based on the probe positioning on the tongue as well as tongue thickness [27]. Additionally, variations in SpO_2_ values have been documented in dogs when using different probe configurations, which encompass placing a half, single, or double thickness of gauze swab between the tongue and the probe. These deviations are likely attributable to differing external pressures applied to the tissue [20]. Especially, in our study, variances in pressure might have resulted from securing the adhesive probe around the folded tongue.

We finally examined ORi in hemodynamically stable dogs. Further studies are necessary to validate the applicability of ORi in critically ill patients who are receiving inotropes, vasopressors, or fluid resuscitation. These treatments could potentially impact the accurately of ORi. In human studies, the accuracy of conventional pulse oximetry has been demonstrated to be influenced by vasoactive drugs [28, 29]. Additionally, the pulse CO-oximeter Radical-7 has exhibited inaccuracies in the non-invasive measurement of haemoglobin concentration in patients with hypoperfusion [30].

Lastly, an *a posteriori* power analysis for linear mixed models [31] was applied to the results because the sample size calculated *a priori* was based on Pearson correlation, that did not account for the repeated pair observation. The analysis returned a power of 100% with a 95% confidential interval between 99.6 and 100%, suggesting that the sample size was appropriate.

## Conclusion

In anesthetized and mechanically ventilated dogs, ORi exhibited a moderate correlation with PaO_2_ across the range of 74 to 258 mmHg. However, it should be noted that this index cannot substitute the blood gas analysis when an accurate measurement of PaO_2_ is clinically relevant. A range of ORi values falling between 0.53 and 0.76 may offer a reasonably dependable indication of PaO_2_ levels from 150 to 190 mmHg. As a result, ORi might be a valuable tool for optimizing oxygen supplementation in dogs.

## Methods

### Animals

After obtaining the owners’ informed consent, 37 adult client-owned dogs of different breeds were enrolled in a prospective observational study approved by the Animal-welfare committee of the University of Padova (OPBA Authorization 55/2021). The study included dogs aging ≥ 10 months, weighing ≥ 14 kg, and classified with an American Society of Anaesthetists risk score (ASA) of I and II. Dogs were admitted to the University Veterinary Teaching Hospital and underwent soft tissue surgeries or diagnostic imaging procedures under general anaesthesia. Animals were enrolled if anaesthesia required the insertion of an arterial catheter for systemic arterial blood pressure measurement and had an unremarkable complete blood test results performed no more than three days before. Dogs with a clinical history of respiratory problems in the previous two months, such as cough, laboured and difficult breathing, or nasal discharge, were excluded.

### Anaesthesia

Food, but not water, was withheld 8 h before anaesthesia. Dogs undergoing diagnostic procedures received butorphanol (Torphedine 10 mg/ml, Dechra Veterinary Products S.r.l.) 0.1–0.2 mg/kg alone or mixed with dexmedetomidine 2–4 µg/kg (Dexdomitor 0.5 mg/mg, Vétoquinol Italia S.r.l.) intramuscularly as premedication before intravenous catheter placement. An animal also received midazolam 0.1 mg/kg (Midazolam 5 mg/ml, B. Braun Milano S.p.A). In patients undergoing surgical procedures, methadone (Semfortan 10 mg/ml, Eurovet Animal Health B.V.) was administered instead of butorphanol for pre-emptive analgesia. General anaesthesia was induced with propofol (PropoVet 10 mg/ml, Zoetis) 2–5 mg/kg intravenously. Following orotracheal intubation, anaesthesia was maintained with sevoflurane (Sevorane, AbbVie S.r.l.) or isoflurane (Isoflo, Zoetis) carried in a mixture of oxygen and air to obtain a FiO_2_ between 0.21 and 0.50. Dogs were mechanically ventilated under pressure-control mode with a peak inspiratory pressure of 10–12 cmH_2_O and an apparatus intrinsic positive end expiratory pressure of 2–3 cmH_2_O [32] The respiratory rate was adjusted to maintain an end-tidal pressure of carbon dioxide (EtCO_2_) between 35 and 45 mmHg. A constant rate infusion of ketamine 0.01–0.02 mg/kg (Nimatek 100 mg/ml, Dechra Veterinary Products S.r.l.) provided perioperative analgesia as needed. The anaesthetist in charge of the case decided the anaesthetic protocol and the dose of drugs according to the guideline of good clinical practice. In dogs scheduled for magnetic resonance imaging, monitoring included direct blood pressure [systolic (SAP), mean (MAP), and diastolic (DAP) pressure], EtCO_2_, and FiO_2_. Other procedures included additionally an electrocardiogram and an oesophageal temperature as basic monitoring. A multiparameter monitor (Datex S/5; GE Healthcare; Helsinki, Finland) continuously displayed those parameters. A multi-wave pulse CO-oximeter (Rad-97, Masimo Corp., CA, USA) constantly showed the pulse wave and the SpO_2_. Lactate Ringer’s solution (B. Braun, Italy) 3–5 ml/kg/h was infused IV during the procedure. The arterial catheter 22G (Denta Med, Italy) was aseptically inserted into the dorsal pedal or palmar artery after anaesthesia induction for blood pressure measurement and blood collection. A mean blood pressure below 65 mmHg was treated by decreasing the volatile agent; if pressure did not improve after 15 min, a 5 ml/kg crystalloid bolus was infused over 10 min. Inotropes or vasopressors were administered if the previous treatments did not increase the pressure. If a dog received one of those treatments, no further data were collected from that subject until 30 min had elapsed from the end of the treatment.

### ORI measurements and arterial blood sample collection

The multi-wavelength pulse CO-oximeter used to measure the SpO_2_, displayed also ORi and PI. The adhesive sensor (Rainbow Lite SET-1 Neo; Masimo Corp., CA, USA) was wrapped circumferentially around the folded tongue and then connected to the CO-oximetry.

The arterial partial pressure of oxygen (PaO_2_) was measured by a blood gas analyser (EDAN i15, EDAN, China) using a multi-parameter cartridge (Test Cartridge BG10, EDAN, China). The blood sample, collected through the arterial catheter into a pre-heparinized syringe (Pulset; Westmed Inc., AZ, USA) over two to three breaths, was analysed immediately after collection. Two ml of blood were drawn and discarded to avoid sample contamination or dilution, and after sampling the artery was flushed with 1 ml of heparinized solution (10 UI/ml).

All measurements were recorded during the anaesthesia maintenance. There were no fixed time points for data collection, and consecutive measurements were separated by at least a FiO_2_ difference of 0.05. Data collection started after a 10-minute equilibration period of stable inspired oxygen concentration after each change in FiO_2_. The ORi was recorded if the heart rate matched the pulse rate recorded by the CO-oximeter and the pulse wave displayed was stable for at least 2 min. At the time of arterial blood sampling, SpO_2_, ORi, pulse rate (PR), PI, oesophageal temperature (Temp), FiO_2_, EtCO_2_, were recorded. Blood variables measured were: temperature-corrected blood pH, temperature-corrected PaO_2_, temperature-corrected arterial blood carbon dioxide (PaCO_2_), haematocrit (Hct), lactatemia (lac), bicarbonate (HCO_3−_), and calculated total haemoglobin (Hb).

The pulse CO-oximeter probe was not repositioned between consecutive paired measurements in the same dog.

### Statistical analysis

Based on the results of a study conducted on donkeys [12], an estimated sample size of 83 paired measurements was able to detect a Pearson’s correlation coefficient (r) of 0.5 between ORi and PaO_2_, with a power of 95% and an alpha of 0.01 and a 25% drop-out rate. All statistical analysis was performed using RStudio (RStudio, PBC, Boston, MA, US) as interface for R (The R Foundation for Statistical Computing, Austria). The Shapiro-Wilk test was used to evaluate the normal distribution of continuous parameters. Normally distributed data were expressed as mean ± standard deviation, while non-normally distributed values as median (minimum-maximum).

The correlation between ORi and PaO_2_ was analysed using a linear mixed-effects regression model for repeated measurements. The fixed effect in the model were PaO_2_, as primary variable of interest, and included PI, Hct, and PaCO_2_ as confounding factors. To account for within-subject correlation across the repeatedly measured data, we introduced dogs as random effects [33]. “Trending ability” defined as the ability of ORi to identify dependable and proportional changes of PaO_2_, was evaluated with the 4-quadrant plot. The analysis was also used to calculate the sensitivity and specificity of ORi in predicting PaO_2_ changes, as reported previously [11]. Briefly, the delta of PaO_2_ between consecutive blood samples was plotted against the delta of the corresponding measurements of ORi. The percentage of data points in the upper right and lower left quadrants over the data points in all quadrants represent the concordance rate. A concordance rate over 90% was judged indicative of good trending ability [34].

The optimal cut-off point to predict a PaO_2_ ≥ 150 mmHg and PaO_2_ ≥ 190 mmHg was determined using Youden’s index, which aims to maximize the sum of sensitivity and specificity while assigning equal weight to false-positive and false-negative results [35]. The test retuned also the area under the receiver operating characteristic curve. The effects of PI, Hb, pH and PaCO_2_ on the diagnostic performance of ORi in predicting PaO_2_ ≥ 150 mmHg were estimated using the semiparametric approach described by Farragi [36].

## Data Availability

All data are included in this published article. The raw datasets are available from the corresponding author on reasonable request.

## List of Abbreviations

BCS: Body condition score
FiO_2_: Inspired fraction of oxygen
Hb: Haemoglobin
HCO_3_^−^: Bicarbonate
SAP: Systolic arterial blood pressure
MAP: Mean arterial blood pressure
DAP: Diastolic arterial blood pressure
ETCO_2_: End-tidal pressure of carbon dioxide
Hct: Haematocrit
Lac: Lactate
ORi: Oxygen Reserve index
PaCO_2_: Arterial partial pressure of carbon dioxide
PaO_2_: Arterial partial pressure of oxygen
PI: Perfusion index
r^2^: Coefficient of determination
SaO_2_: Arterial oxygen saturation
SpO_2_: Peripheral oxygen saturation
SvO_2_: Venous oxygen saturation
